# Little Girl

**Published:** 1874-12

**Authors:** 


Little Girl—"Mamma, I don't think the people who make dolls are very pious people." Mamma—"Why not, my child ?" Little Girl—"Because you can never make them kneel. I always have to lay my doll down on her stomach to say her prayers."

